# Quantification of Gender Bias and Sentiment Toward Political Leaders Over 20 Years of Kenyan News Using Natural Language Processing

**DOI:** 10.3389/fpsyg.2021.712646

**Published:** 2021-12-10

**Authors:** Emma Pair, Nikitha Vicas, Ann M. Weber, Valerie Meausoone, James Zou, Amos Njuguna, Gary L. Darmstadt

**Affiliations:** ^1^Department of Pediatrics, Global Center for Gender Equality, School of Medicine, Stanford University, Stanford, CA, United States; ^2^Department of Neuroscience, University of Texas – Dallas, Dallas, TX, United States; ^3^School of Public Health, University of Nevada, Reno, NV, United States; ^4^Research Computing Center, Stanford University, Stanford, CA, United States; ^5^Department of Biomedical Data Science, Stanford University, Stanford, CA, United States; ^6^School of Graduate Studies, Research and Extension, United States International University - Africa, Nairobi, Kenya

**Keywords:** artifical inteligence, natural language processing (NLP), gender bias, gender equality, leadership, sentiment, Kenya, political

## Abstract

**Background:** Despite a 2010 Kenyan constitutional amendment limiting members of elected public bodies to < two-thirds of the same gender, only 22 percent of the 12th Parliament members inaugurated in 2017 were women. Investigating gender bias in the media is a useful tool for understanding socio-cultural barriers to implementing legislation for gender equality. Natural language processing (NLP) methods, such as word embedding and sentiment analysis, can efficiently quantify media biases at a scope previously unavailable in the social sciences.

**Methods:** We trained GloVe and word2vec word embeddings on text from 1998 to 2019 from Kenya’s *Daily Nation* newspaper. We measured gender bias in these embeddings and used sentiment analysis to predict quantitative sentiment scores for sentences surrounding female leader names compared to male leader names.

**Results:** Bias in leadership words for men and women measured from *Daily Nation* word embeddings corresponded to temporal trends in men and women’s participation in political leadership (i.e., parliamentary seats) using GloVe (correlation 0.8936, *p* = 0.0067, *r*^2^ = 0.799) and word2vec (correlation 0.844, *p* = 0.0169, *r*^2^ = 0.712) algorithms. Women continue to be associated with domestic terms while men continue to be associated with influence terms, for both regular gender words and female and male political leaders’ names. Male words (e.g., he, him, man) were mentioned 1.84 million more times than female words from 1998 to 2019. Sentiment analysis showed an increase in relative negative sentiment associated with female leaders (*p* = 0.0152) and an increase in positive sentiment associated with male leaders over time (*p* = 0.0216).

**Conclusion:** Natural language processing is a powerful method for gaining insights into and quantifying trends in gender biases and sentiment in news media. We found evidence of improvement in gender equality but also a backlash from increased female representation in high-level governmental leadership.

## Introduction

### Kenyan Political Landscape

Following the outbreak of violence after its 2007 elections, Kenya adopted a new constitution in 2010 – a step forward for Kenyan gender equality. The constitution restructured power by devolving authority and resources to 47 newly created counties. Constitutional Article 81(b) stipulated that no more than two-thirds of the members of elected public bodies should be of the same gender, so 47 seats in the National Assembly were set aside for women. At the county level the constitution provides a mechanism to achieve the two-thirds gender rule and most counties are in compliance (Berry et al., 2020). However, attempts to pass legislation to achieve this quota at the national level and create a mechanism for reaching it have failed, as only 22 percent of the members of the 12th Parliament inaugurated in 2017 were women (Inter-Parliamentary Union [IPU], 2020).

The Political Parties Act of 2011 mandated that parties cannot be registered or receive financial support from the political party fund unless they ensure gender equality, but there has been weak enforcement of this regulation (Tripp et al., 2014). The expected balancing in their governing structures and allocation of at least one-third of their funds towards promoting female candidates has not occurred (Tripp et al., 2014). The low level of women’s representation in national political leadership is due in part to a lack of political will on the part of politicians and political parties (Kivoi, 2014; National Democratic Institute and Federation of Women Lawyers [FIDA] Kenya, 2018), lack of enforcement of legislation, failure by parties to comply with the regulations guiding political party primaries, limited financial resources of candidates, and socio-cultural stigmatization and gender-based electoral violence (Kivoi, 2014; Tripp et al., 2014; Tundi, 2014; National Democratic Institute and Federation of Women Lawyers [FIDA] Kenya, 2018).

Cultural, financial and political factors appear to act together as barriers to potential female political leaders (Kivoi, 2014; Tundi, 2014; National Democratic Institute and Federation of Women Lawyers [FIDA] Kenya, 2018), including backlash from the entrenched male elite in response to women’s entry into this male-dominated space (Tripp et al., 2014; Berry et al., 2020). Due to patriarchal structures in African society, women in leadership are placed secondary to men in prevailing customs and culture (Kiamba, 2008). In Kenya, perceptions of women as subordinate to men continue as many people uphold cultural practices, such as child marriage and delegation of women to domestic roles (Kasomo, 2012; Kivoi, 2014; Tundi, 2014). It may be considered a sign of disrespect for a woman to express her opinion in the presence of men as men consider women’s ideas as inferior (Tundi, 2014).

### Gender in the Media

Investigating trends in gender bias is necessary to better understand the gender landscape in Kenya and address the current political blockade on proper legislation and its enforcement for gender equality. Analyzing bias in the media is one way to accomplish this. Media has an important influence on political attitudes and potentially electoral outcomes (Druckman and Parkin, 2005; Tripp et al., 2014). The media can also set the agenda by determining which issues are important to report, indicated by placement or amount of coverage, and influencing the importance audiences give the issues and ultimately, policy outcomes (McCombs and Shaw, 1972). In an analysis of Kenyan newspapers, Ochieng (2018) found that both the *Daily Nation* and *Standard* newspapers gave little coverage to the constitutional implementation of the two-thirds gender rule during the electioneering period of 2017 and therefore failed to set the two thirds gender rule as a prominent enough issue (Ochieng, 2018).

### Analysis of Gender Bias Using Natural Language Processing

Traditional social science research analyzing bias in news texts has relied on cumbersome manual methods of analysis such as content analysis and framing analysis (Hamborg et al., 2019). In contrast, natural language processing (NLP) methods are able to rapidly generate summaries on vastly larger amounts of data. They offer a way to quantify gender bias in the media at a scope and speed not available in traditional social science analyses.

Word embedding is a mathematical model typically used in NLP that maps a set of words or phrases in a vocabulary to vectors of numerical values. These models have been found to encode meaningful semantic relationships among words, such as capturing gender biases in analogies. For example, using Mikolov’s word2vec embedding trained on a Google News dataset, Bolukbasi et al. (2016) found that word embedding returned “homemaker” for the analogy “man is to computer programmer as woman is to x” (Bolukbasi et al., 2016). It has been shown that biases found in word embeddings trained on news corpora reflect societal gender bias in additional ways, such as mimicking results found in the Implicit Association Test, with women being more associated with the arts than sciences (Bolukbasi et al., 2016). The Implicit Association Test is meant to measure the strength of associations between concepts (e.g., women, men) and evaluations (e.g., good, bad) or stereotypes. Caliskan et al. (2017) and Garg et al. (2018) also demonstrated that word embeddings reflect historical gender differences in occupations in labor force participation.

Sentiment analysis has become an increasingly prevalent method to analyze media by categorizing text as positive, negative, or neutral (Pang and Lee, 2008). Methods of sentiment analysis include lexicon-based approaches, which use a dictionary of words (labelled as positive or negative) to match sentiment in an input text, or machine learning-based approaches (Verma and Thakur, 2018). Few sentiment analysis studies of media have investigated gender differences in media and social network communications. In this study, we used a novel application of sentiment analysis to examine the portrayal of female vs. male leaders within one media source, Kenya’s *Daily Nation* newspaper, by separating and comparing sentences that include male vs. female leader names. We present an advanced machine-learning based approach to quantify sentiments per sentence.

Most research on gender bias in word embeddings has been done with American text sources, such as Wikipedia common crawl, Google News, *New York Times* text and other English news sources. We investigated whether bias in word embeddings trained on Kenyan news text reflect temporal trends in gender bias in Kenyan society. Further, we used sentiment analysis to analyze temporal trends in portrayal of female and male leaders in Kenya. Unlike many works that investigate sentiment analysis or word embeddings separately or use word embeddings as a numerical input for classification tasks like sentiment analysis, we investigate them both individually, but incorporate findings from both into a more comprehensive portrayal of the text. This work addresses the call for more investigation into gender representation in African news media (Gadzekpo, 2009; Bosch, 2011) and media in general (Global Media Monitoring Project, 2015). We hypothesized that bias identified in our word embeddings would mirror Kenyan historical gender trends and also be reflected in sentiment analysis, and thus would open up new ways of exploring gender bias at a societal level.

## Materials and Methods

### Data

For data on historical trends in men and women’s participation in political leadership in Kenya, we used the Inter-Parliamentary Union’s (IPU) publicly available archive of the percentage of men and women in Kenyan Parliament (Inter-Parliamentary Union [IPU], 2020). To understand trends in popular Kenyan opinion on gender, we used the Afrobarometer’s online analysis tool (Afrobarometer, 2020). We utilized the survey question, “Which of the following statements is closest to your view? Choose Statement A, or, Statement B. A: Women should have the same chance of being elected to political office as men. B: Men make better political leaders than women and should be elected rather than women.”

### The *Daily Nation*

The *Daily Nation* is the most widely read Kenyan newspaper (Answers Africa, 2020). It was purchased by Nation Media Group (NMG) Limited in 1959, which is now the largest independent media house in East and Central Africa (Maina, 2006). Despite some pro-government tendencies and history, it is generally considered to be the newspaper with the most balanced reporting in Kenya (Maina, 2006).

### Word Embedding Analysis

#### Bias and Historical Trend Analysis

Two common word embedding algorithms include GloVe (Pennington et al., 2014) and word2vec (Mikolov et al., 2013). Word2vec is considered a local context window method, capturing co-occurrence information one window at a time, while GloVe is a weighted least squares model that trains on global word-word co-occurrence counts (Pennington et al., 2014). There are conflicting findings on their comparative performance, but similar to Levy we expect them to perform similarly (Levy et al., 2015).

**FIGURE 1:**
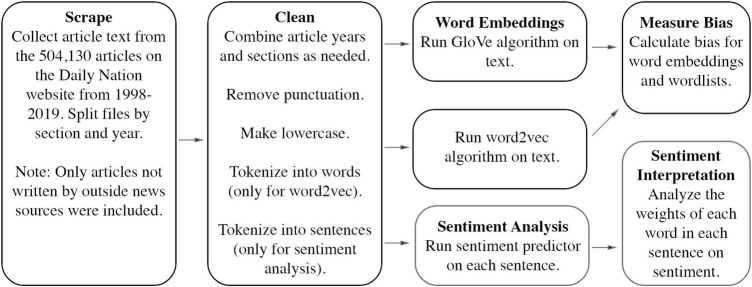
Schematic of word embedding and sentiment analysis methodology used to measure gender bias in Kenya’s *Daily Nation* newspaper.

For our temporal analysis of gender bias in Kenyan society, we used GloVe embeddings trained on all news article text available online from the *Daily Nation* from 1998 to 2019 (Figure 1). We collected text for each article in each section (News, Counties, Business, Opinion, Sports, and Lifestyle) for each year, excluding articles written by international groups such as Agence France-Presse. We also combined the text from individual years into groups of three consecutive years (e.g., 1998-2000, 2001-2003) and created embeddings for each grouping. As a check on the quality of the GloVe three-year embeddings, we also created GloVe embeddings for each separate year for comparison. All text was moved to lower case, and punctuation and numbers were removed. Our embeddings were all 150 dimensions (the vectors were 150 in length). As a further check on the consistency of our embeddings, we used the same text to train embeddings using a word2vec algorithm.

Next, we created several wordlists to test for gender bias in the word embeddings. We created wordlists to represent gender and other wordlists that are not inherently associated with men or women (neutral word lists) to measure the association of men and women with certain concepts, such as leadership. We used the same wordlist as Garg et al. (2018) for female and male words and created a neutral leader wordlist (words related to leaders but not specifically to men or women) and neutral wordlists for adjectives related to leadership (which can be applied to men or women in positions of leadership) (Table 1). We note that we could only make gender binary in our analysis but acknowledge that gender exists on a spectrum.

**TABLE 1:** Wordlists for male and female gender words, names of male and female leaders, neutral leader words, and neutral adjectives related to leaders.

Category	Wordlist
**Gender words**	

Female words^1^	she, daughter, daughters, hers, her, mother, woman, girl, herself, female, sister, sisters, mothers, women, girls, females, aunt, aunts, niece, nieces

Male words^1^	he, son, his, him, father, man, boy, himself, male, brother, sons, fathers, men, boys, males, brothers, uncle, uncles, nephew, nephews

**Names (first or last name) of male and female leaders**	

Female leader names	anne, waiguru, martha, karua, charity, ngilu, nancy, baraza, ann, ngirita, philomena, mwilu, gladys, shollei, susan, kihika, orie, rogo, manduli, esther, muthoni, passaris, margaret, wanjiru, millie, odhiambo, racheal, ruto, njoki, ndungu, gladys, wanga

Male leader names	uhuru, kenyatta, daniel, toroitich, arap, moi, william, ruto, raila, odinga, mwai, kibaki, kalonzo, musyoka, moses, wetangula, aden, duale, musalia, mudavadi, mike, mbuvi, sonko, evans, kidero, gideon, james, orengo, john, michuki, kiraitu, muriungi

**Leader words (neutral with respect to gender)**	

Leaders	president, presidents, minister, ministers, leader, leaders, leadership, director, directors, officer, officers, chief, chiefs, authority, authorities, executive, executives, manager, managers, boss, bosses, politician, politicians, mayor, mayors, captain, captains, premier, premiers, governor, governors, commander, commanders, supervisor, supervisors

**Adjectives related to leaders (neutral with respect to gender)**	

Competence	competent, qualified, knowledgeable, accomplished, proficient, skilled, adept, practiced, experienced, expert, capable, able, skillful, prepared, credible, suitable, efficient, resourceful, educated, informed, professional, trained, talented, gifted, masterly, equipped, suited, masterful, pro, fit, ready, ace, master, schooled, overqualified, licensed, adequate, versed, employable, virtuoso, informed, erudite

Intelligence	intelligent, smart, clever, bright, shrewd, wise, sharp, genius, brilliant, discerning, canny, resourceful, intuitive, insightful, savvy, sagacious, adaptable, astute, keen, brainy, inventive, creative, imaginative, perceptive, intellectual, learned, observant, crafty, witty, educated, highbrow, original, ingenious, artful, sage, agile, quick, innovative, alert, cerebral, foresight

Rationality	reasonable, sensible, rational, responsible, practical, strategic, diplomatic, calm, logical, agreeable, composed, prudent, pragmatic, realistic, reasoning, sane, calculating, politic, proper, commonsense, stable, mindful, cool, sober, grounded, analytical, judicious, tactical, tactful, levelheaded, aware, lucid, clearheaded, cautious, utilitarian, opportunistic, sapient, sound, analytic, justified, civil

Influence	influential, powerful, power, influence, confident, confidence, visionary, vision, strong, inspiring, engaging, charismatic, magnetic, respected, inspirational, esteemed, important, prominent, potent, significant, dominant, authoritative, leading, impressive, persuasive, forceful, eminent, notable, effective, charisma, famous, compelling, mighty, distinguished, famed, renowned, robust, convincing, foremost, commanding

Good leader	considerate, virtuous, courteous, good, altruistic, integrity, trustworthy, accountable, transparent, humble, empathetic, positive, courageous, optimistic, bold, loyal, selfless, truthful, genuine, principled, best, straightforward, honorable, fair, sincere, moral, kind, benevolent, respectable, just, upright, honest, ethical, noble, thoughtful, respectful, excellent, upstanding, righteous, responsible, heroic, brave, valiant

Determination	determined, dedicated, committed, purposeful, persevering, ambitious, devoted, unwavering, steadfast, caring, faithful, diligent, industrious, steady, resolute, firm, decided, tenacious, resolved, unshakeable, persistent, certain, tireless, spirited, decisive, constant, dogged, intentional, serious, staunch, aspiring, enterprising, focused, solid, intent, devout, dutiful, unfaltering, patient, assured

Immoral	loose, immoral, prostitute, indecent, lewd, lustful, promiscuous, wanton, racy, immodest, dirty, careless, nasty, inappropriate, filthy, improper, vulgar, sinful, wicked, depraved, vile, shameless, impure, shameful, lecherous, ungodly, carnal, obscene, raunchy, crude, provocative, scandalous, sleazy, wretched, trashy, profane, raunchy, bawdy, foul, smutty

Corruption	corruption, duplicity, corrupted, immoral, bribe, bribes, bribed, bribery, fraud, fraudulent, manipulate, corrupt, nefarious, shady, unethical, crooked, untrustworthy, exploit, dishonest, dishonesty, crime, graft, extortion, nepotism, theft, misappropriation, thief, irregularities, irregular, fictitious, wrongdoing, robbery, crookedness, injustice, exploitation, criminal, criminality, unlawful, illegitimate, felony, felonies, debauchery, crime

Bad leader	authoritarian, authoritarian, tyrannical, tyranny, tyrant, cruel, cruelty, dictatorial, dictator, dictatorship, autocratic, totalitarian, repressive, ruthless, oppressive, undemocratic, corrupt, tribal, violent, violence, rude, inciter, chaos, chaotic, difficult, cronyism, bossy, oppressor, bully, evil, warlord, inhumane, brutal, hateful, malevolent, sadistic, savage, hostile, instigator, despot, despotic

Defamation	scandal, slander, libel, defamed, smear, stigma, discredited, mudslinging, vilification, insult, defamation, allegations, scam, tarnished, criticized, criticism, rumor, calumny, disparagement, defame, insulted, defamatory, discredit, gossip, belittlement, dirt, accusation, accusations, dishonour, debasement, shame, disparaged, vilified, denigrated, malign, allegation, dishonored, debased, shamed, demeaned

**Adjectives related to gender roles**	

Domestic	homemaker, domestic, rearing, childrearing, housekeeping, parenting, cleaning, childcare, household, caregiving, errands, children, caregiver, babysitting, home, laundry, cooking, washing, caretaker, kitchen, housework, family, chores, house, scrubbing, tidying, sweeping, scrub, sweep, cook, babysit, wash, child, chore, dusting, ironing, mopping, mop, iron, sewing, sew, schoolwork, errand, housekeeper, maid, househelp

*^1^Male and female words are from Garg et al. (2018).*

Using the same methodology as Garg et al. (2018), we measured the strength of the association between neutral leader words and gender words. First, we created a vector representing the female noun wordlist by averaging the vectors for each word in this list. We did the same for the male noun wordlist. Next, we measured the cosine similarity between each vector (representing one word) in the neutral leader wordlist and the gender word vector and averaged them all to derive the association between the neutral leader word list and the gendered noun word list. Cosine similarity is a common approach for measuring this association (Bolukbasi et al., 2016; Caliskan et al., 2017; Kozlowski et al., 2019). We subtracted the association with men from the association with women to derive the bias. If the bias value was negative, then the embeddings associated men more with neutral leader words than women. Conversely, if the bias value was positive, then the embeddings associated the neutral leader words with women more than men.

To determine whether the embedding bias reflected gender biases in Kenyan society, we compared our embedding bias to historical trends for men and women in political leadership based on IPU data. Specifically, we examined the trend in the percentage difference between male and female seats in parliament.

#### Leader Qualities Bias Analysis

We created lists of adjectives that are neutral with respect to gender but that represent various qualities of leaders, including positive traits (e.g., competence, intelligence, rationality, influence, good leader, and determination), negative traits (e.g., immoral, corruption, bad leader, defamation) and a wordlist that represents domestic work (Table 1). We measured the gender bias over time associated with these wordlists of adjectives related to leaders using the same methodology as we did with leader words. To determine if there was a difference in the use of male and female gender words compared to specific male and female leaders, we created a list of the first and/or last names of male and female political leaders to act as another set of gender-related wordlists (Table 1).

We used Pearson correlations to measure the linear trend between bias trends and historical trends. We used ordinary least-squares regressions to measure linear trends in word embedding bias. Trends with *p* ≤ 0.05 were considered significant.

### Sentiment Analysis

Sentiment analysis was conducted to compare the sentiments of sentences associated with female leaders (i.e., the names of specific female leaders) to those associated with male leaders in the *Daily Nation* from 1998 to 2019. Sentences that included an element in the male and female leaders names lists, as well as the sentences immediately before and after, were filtered and separated to represent the media’s presentation of female and male leaders.

For our temporal sentiment analysis, we used an established sentiment analysis method to predict a sentiment score for each sentence. Specifically, we used a sentiment prediction model implemented in the AllenNLP library (Gardner et al., 2018; Hagiwara, 2020) and trained it on the Stanford Sentiment Treebank (Socher et al., 2013). Annotated Kenyan text was not available for training the sentiment analysis model, which may be a limitation of the model. The predictor determines a sentiment score ranging from 0 (very negative) to 4 (very positive) for each sentence. For each year and gender, distributions of the five sentiment scores were calculated. The percentage of negative sentences with scores of 0 or 1 in the text was graphed for each year and each gender. We subtracted the percent negative of the male text from the percent negative of the female text to obtain a gender-difference in sentiments for each year. If the difference was negative, the male text had a higher percentage of negative sentiments than the female text. These steps were repeated for the percent of positive sentences with scores of 3 or 4. A simple linear regression was used to assess the change in the difference between negative and positive sentiments associated with female and male leaders over time.

To gain a better understanding of the model’s performance on Kenyan English, a sample of *Daily Nation* sentences were hand-labelled and compared to the predictor’s scores. Fourty sentences from each gender and time period combination were randomly selected, for a total of 560 sentences. These sentences were hand labelled as positive, negative, or neutral by two scorers, achieving agreement. Using these labelled values and model-predicted scores, the accuracy, precision, recall, and f1 were computed.

## Results

### Word Embedding Bias and Historical Trends in Political Leadership

**FIGURE 2:**
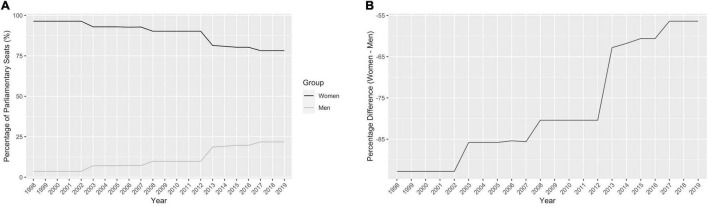
The percentage of men and women in Kenyan parliament. **(A)** The percentage of men and women that made up the Kenyan Parliament from 1998 to 2019, Kenya Inter-Parliamentary Union data (Inter-Parliamentary Union [IPU], 2020). **(B)** The difference in percentage (women – men) between men and women in the Kenyan Parliament from 1998 to 2019.

The percentage of men and women that made up the Kenyan Parliament from 1998-2019 is shown in Figure 2A. The absolute difference in the percentage of men and women in parliamentary seats (male percentage subtracted from female percentage) decreased between 2002 and 2017 (Figure 2B). The sharpest increase of women in parliamentary seats occurred from 2012 to 2013, which corresponds with the adoption of the two-thirds gender rule in the new 2010 Kenyan constitution.

**FIGURE 3:**
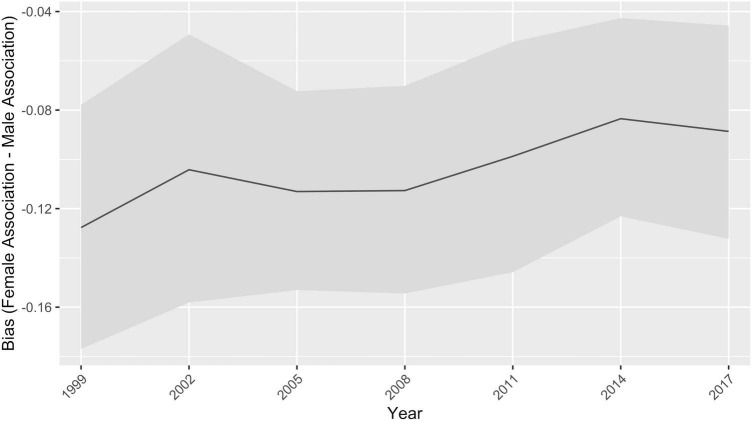
Three-year bias for leader words related to gender words. A negative bias means the leader word list is more associated with men. 95% confidence intervals are shown.

Over time, bias for gender words related to leader words became less negative, or became more equal, correlating with the historical trend in parliamentary seats (three-year GloVe word embedding correlation = 0.8936, *p* = 0.0067, *r*^2^ = 0.799) (Figure 3). Thus, the GloVe word embeddings correlated with historical gender trends in political leadership, with women increasingly associated more with being a leader and occupying seats in parliament. Similar results associating bias for words related to women and men as leaders with historical trends in parliamentary positions were found using one-year GloVe word embeddings (correlation = 0.737, *p* = 9.24e-05, *r*^2^ = 0.543) (Supplementary Appendix 1). For word2vec vectors, the correlation over time between bias in leader words – becoming less negative, or more associated with women over time – and

increased percentage of parliamentary seats held by women also was significant (correlation 0.844, *p* = 0.0169, *r*^2^ = 0.712).

### Gender-Based Surveyed Attitudes Toward Leadership

**FIGURE 4:**
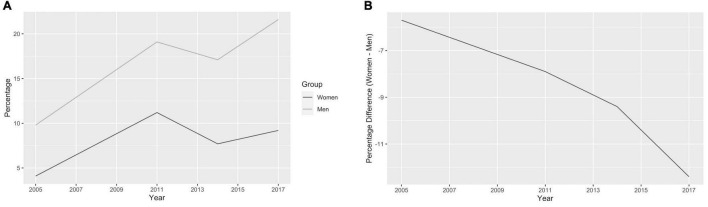
Afrobarometer survey results on gender and leadership. **(A)** The percentage of women and men who “strongly agree” that men make better leaders than women (from the 2005-2017 Afrobarometer surveys) (Afrobarometer, 2020). **(B)** The difference between the percentage of women and men who “strongly agree” that men make better leaders than women.

To understand societal-level beliefs on women in leadership, we examined trends in the Afrobarometer survey results for Kenya. We found that across years both women and men increasingly “strongly agree” that men make better leaders but the percentage of male respondents agreeing that men make better leaders has increased more quickly than it has for female respondents (Figures 4A,B). Results were similar for urban and rural men, but a higher percentage of rural women than urban women (∼2-3%) agree that men make better leaders (Supplementary Appendix 2). Education level did not make a difference except as reflected in data for men with informal schooling; in 2005, 18.8% of men with informal schooling strongly agreed men made better leaders, whereas in 2016, this percentage was 47.5% (Supplementary Appendix 2).

### Gender Representation in the *Daily Nation*

Male words were mentioned 1.84 million more times than female words in the *Daily Nation* from 1998 to 2019. The per year numerical difference between mentions of men and women has increased since 1998. It peaked at 109,881 more mentions of men than women in 2013 and has stayed relatively constant since (Supplementary Appendix 3).

### Word Embedding Gender Bias in Qualities of Leaders

**FIGURE 5:**
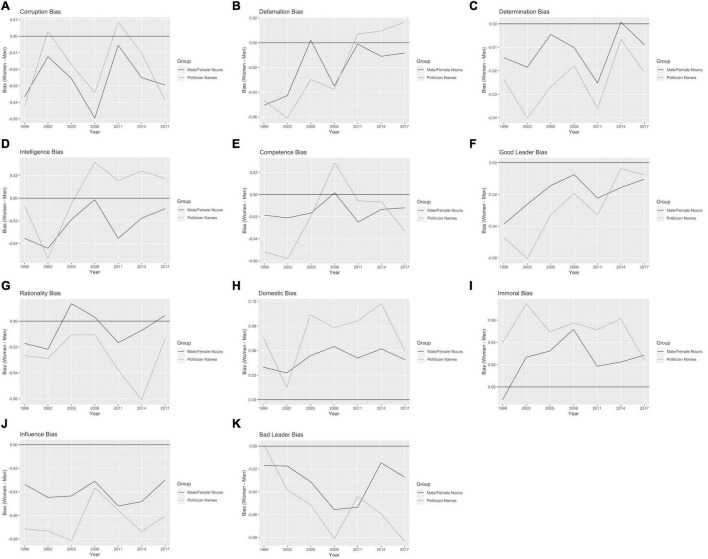
Bias for gender words and leader names and word lists for descriptors related to leadership. For **(A)** Corruption; **(B)** Defamation; **(C)** Determination; **(D)** Intelligence; **(E)** Competence; **(F)** Good leader; **(G)** Rationality; **(H)** Domestic; **(I)** Immorality; **(J)** Influence; and **(K)** Bad Leader. Note that the defamation terms could be associated with the individual defamed or the individual doing the defaming (if there was one).

Associations of gender words (i.e., female nouns, male nouns) bias trends or leader names (male names, female names) bias trends with adjectives related to qualities of leaders were mixed (Figures 5A-K). In general, domestic terms (Figure 5H) and immorality terms (Figure 5I) were associated more with women than with men over the study period. In contrast, terms for influence (Figure 5E) continued to be associated more with men than women. Good leader adjectives were associated more with men than women, but became less associated with men over time (Figure 5F). These findings held true for three-year gender words as well as three-year leader names. Terms for bad leaders (tyrannical, violent) (Figure 5K) became more associated over time with male leaders names and were continuously associated with male words, but this pattern was variable and overall showed no significant trend. See Supplementary Appendix 4 for one-year bias graphs.

No wordlists for gender words had significant linear trends for adjectives of leader qualities with three-year word embeddings. Wordlists for leader qualities that had significant linear trends (*p* ≤ 0.05) for three-year word embedding bias for male and female leader names included good leader terms (*p* = 0.011, slope = 0.0028, associated with men but trending toward women), bad leader terms (*p* = 0.0418, slope = −0.0034, associated with men and increasing over time) and defamation terms (*p* = 0.0036, slope = 0.0044, associated with men but trending toward women and in 2011 became more associated with women) (Table 2A). Trends associating bias for male and female leader names with all other leader adjectives were non-significant (Table 2A).

**TABLE 2A:** Slopes and trends for all wordlists using three-year word embeddings.

Wordlist	Gender words slope^a^	Gender words *p*-value	Gender words associated with	Leader names slope	Leader names *p*-value	Leader names associated with
Competence	0.0003226	0.598	men	0.002053	0.319	men (except 2008^b^)
Intelligence	0.0013707	0.183	men	0.002932	0.103	men then women in 2008
Rationality	0.0007588	0.412	men (except 2005, 2008, 2017)	−0.0006234	0.631	men
Influence	−0.00005827	0.953	men	0.0006843	0.521	men
Good leader	0.0011675	0.0758	men	0.0027575	0.0110*	men
Determination	0.000406	0.508	men	0.0007941	0.301	men
Immoral	0.001163	0.377	women (except 1999)	−0.0008445	0.454	women
Domestic	0.0009960	0.188	women	0.001749	0.464	women
Corruption	0.0001927	0.855	men	0.00013	0.930	men (except 2002, 2011)
Bad leader	−0.0005579	0.650	men	−0.003404	0.0418*	men
Defamation	0.002229	0.0954	men	0.0043834	0.00360**	men then women in 2011

*^a^A positive slope means that the words became more associated with women over time.*

^b^2008 is an average of 2007-2009. This is true for the other years – they represent the average of that year and the year before and after. *p ≤ 0.05, **p ≤ 0.01. Statistically significant p-values are shown in bold print.

Wordlists for leader qualities that had significant linear trends for one-year embedding bias for gender words included domestic terms (*p* = 0.00725, slope = 0.00152, associated with women and increasing over time), and defamation terms (*p* = 0.00255, slope = 0.00247, associated with men but trending toward women and in 2012 became associated with women) (Table 2B. Terms that had significant linear trends for one-year embedding bias for male and female leader names included competence terms (*p* = 0.00526, slope = 0.00213, associated with men but trending toward women and in 2009 became associated with women), good leader terms (*p* = 0.0107, slope = 0.00195, associated with men but trending toward women and in 2015 became associated with women), domestic terms (*p* = 0.0186, slope = 0.00282, associated with women except for in 2001 and 2003 and increasing over time), corruption terms (*p* = 0.0108, slope = 0.00264, associated with men but trending toward women and in 2015 became associated with women), and defamation terms (*p* = 3.26e-05, slope = 5.137e-03, associated with men but trending toward women and in 2012 became associated with women). No other bias trends were significant. See Supplementray Appendix 5 for full regression results.

**TABLE 2B:** Slopes and trends for all wordlists using one-year embeddings.

Wordlist	Gender words slope	Gender words *p*-value	Gender words associated with	Leader names slope	Leader names p-value	Leader names associated with
Competence	0.0004674	0.159	men (except 2009)	0.0021318	0.00526**	men (except 2009, 2010, 2015)
Intelligence	−0.0003707	0.319	men	0.0003688	0.566	neutral
Rationality	−0.0001759	0.581	men (except 1998, 2014)	−0.0005278	0.403	men (except 1998, 2018)
Influence	0.0002747	0.464	men	0.0012902	0.126	men
Good leader	0.0005731	0.135	men	0.0019516	0.0107*	men (except 2015)
Determination	0.0005687	0.122	men (except 2003, 2015, 2018)	0.0014978	0.0874	men (except 2008, 2015, 2017)
Immoral	0.0015625	0.0805	women (except 1998, 1999, 2002, 2008, 2012)	0.002171	0.0777	women (except 1998, 1999, 2008)
Domestic	0.0015218	0.00725**	women (except 2001)	0.002817	0.0186*	women (except 2001, 2003)
Corruption	0.0002045	0.691	men	0.0026373	0.0108*	men (except 2015, 2017)
Bad leader	0.0008556	0.276	men (except 1999, 2015, 2019)	0.0011421	0.152	men
Defamation	0.0024681	0.00255**	men (except 2012, 2013)	5.137e-03	3.26e-05**	men (except 2012, 2013, 2015, 2016, 2017)

*See Supplementary Appendix 5 for full regression results.*

*Statistically significant p-values are shown in bold print.*

### Sentiment Trends for Political Leaders

**FIGURE 6:**
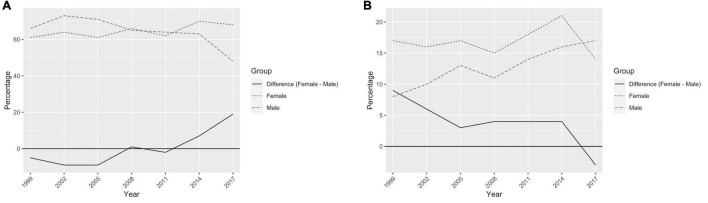
Positive and negative sentiment associated with male and female leader names. **(A)** The percentage of sentences with negative sentiment associated with female or male leader names and the difference in those percentages. **(B)** The percentage of sentences with positive sentiment associated with female or male leader names and the difference in those percentages.

Over time, there was a greater relative increase in the negative sentiment surrounding females compared to males, resulting in a significant increase (*p* = 0.0152, slope = 0.0133) in the difference in percentage of negative sentiments in the text surrounding female leaders compared to male leaders (Figure 6A). Similarly, there was a decrease (*p* = 0.0216, slope = −0.00451) in the difference in percentage of positive sentences in female leader text compared to male leader text, indicating a greater relative increase in the positive sentiment surrounding males compared to females (Figure 6B). Overall, a greater percentage of both female and male sentences each year had negative sentiments (ranging from 48 in 2016 male to 73% in 2001 male) than positive sentiments (ranging from 8 in 1998 to 21% in 2013 female).

When testing the predictor’s accuracy on the sample of 560 hand-labelled sentences, the model performed with an accuracy of 0.59 (weighted precision = 0.59, recall = 0.59, f1 = 0.57).

## Discussion

We aimed to investigate whether trends in word embedding bias correlated with trends in societal gender statistics. We tested the hypothesis that correlations would be identified and thus that NLP methods applied to newspaper text could be used to identify trends in bias related to women and men as political leaders in Kenya. We also further investigated how women in politics were portrayed in the media compared to men using sentiment analysis. Our bias measurements showed some improvement in gender equality in the *Daily Nation* over time as leader bias based on word embedding analysis of the association of leader words with gender words trended towards equality over time, although there appeared to be a dip back towards inequality in 2019. We also found that Parliamentary leadership positions and some adjectives related to qualities of leaders continued to be associated more with men than women over time. This is similar to the finding of Garg et al. (2018) who reported that in the United States, men continued to be associated with occupations more than women but that gender bias decreased over time. Their findings correlated with historical trends in occupations becoming less male-dominated over time.

In our investigation of leader adjectives and other wordlists, we examined trends for both one-year and three-year embeddings. For both embedding types, and for both male and female leader names and male and female words, there was a general consistency in associations of wordlists with men vs. women. For both one-year and three-year embeddings, good leader terms had a significant trend for leader names, with good leader terms more associated with men but becoming increasingly associated with female leaders over time. Men leader names were also associated strongly with defamation in the 2000s but this trended significantly toward women in one-year and three-year embeddings. One-year embeddings showed more statistically significant trends than three-year embeddings, as seen in statistically significant increases in female association with domestic terms for both leader names and gender words and for associations of women leader names with competence and corruption. Differences in the one-year and three-year embeddings could be due to one- year embeddings containing more data points, but because they are created with less text, one-year embeddings are also noisier than three-year embeddings.

We found from the Afrobarometer that the percentage of men and women who agree that men make better leaders increased over time (Afrobarometer, 2020), in the opposite direction as our measurement of bias for gender words related to neutral leader words. Thus, we did not find a correlation between attitude-based bias measures (measures of personal bias) and NLP bias measurements, unlike the finding of Caliskan et al. (2017) for Implicit Association Test results and their measure of bias. Caliskan et al. (2017) showed that their Word-Embedding Association Test, a word-embeddings based statistical test analogous to the Implicit Association Test, replicated the biases found in the Implicit Association Test such as female names being more associated with family than career words compared with male names. This difference between Caliskan’s results and our results may be because the *Daily Nation* may not represent the prevailing attitudes of the public towards women leaders, or at least the attitudes of those surveyed for the Afrobarometer. It appears that our embeddings reflected fact-based historical trends in political representation but not attitudes-based trends.

The strength of word embeddings is that they can capture relationships between words while the strength of sentiment analysis is that it can capture the sentiment of particular sentences or phrases. In our temporal sentiment analysis, we found that negative sentiment sentences were increasingly associated with female political leaders over time while positive sentiment sentences were increasingly associated with male political leaders, though to a lesser extent. There could be many reasons for this. Sentences with negative sentiment were present at a higher percentage than sentences with positive sentiment, indicating that the media generally may offer more of a critical voice than a congratulatory one. The increasing negativity toward women could be a part of a backlash against women entering politics in Kenya after the adoption of the new constitution. This could also be reflected in the increase in the association of domestic terms and defamation terms with women leaders in word embedding analyses. The corresponding positivity toward male leaders in sentiment analysis parallels the increase in the percentage of Kenyans who think men make better leaders seen in the Afrobarometer results (Afrobarometer, 2020). So, while bias against women politicians may be mostly decreasing as they gain exposure related to leadership in the media, the sentiment analysis results and specific word embeddings imply that the way female leaders are presented in the media could be getting more negative over time as they garner more attention from the press. It should also be noted that leadership words are still associated with men. Also, even though leadership terms became more associated with women over time, the embedding bias showed a dip back toward men in both the three-year (in 2017, representing 2016-2018) and one-year (in 2019) embeddings.

Unlike investigators who examined large pre-trained word embeddings, such as Google news or Wikipedia embeddings, we examined word embeddings newly created from a single source, a Kenyan newspaper. We expect these fine-grained embeddings are better able to shed light on trends for the *Daily Nation* itself than a pre-trained embedding, especially one trained on American English instead of Kenyan English. Our embeddings are also likely more suited for analyzing the specific area of leadership and gender in Kenya since they are created from an exclusively Kenyan source. However, we anticipate that larger embeddings created from a variety of Kenyan or East African news sources, like Google News corpora, may more accurately reflect trends of the whole of society, due to the larger amount of text (Pennington et al) (Bolukbasi et al., 2016) and variety of perspectives that multiple news sources would bring. It is more likely that embeddings created from one news source reflects only a portion of the population, and perhaps more specifically that of its readers, writers and editors. Therefore, it is possible that the *Daily Nation* does not fully reflect views of the broader rural population in Kenya. In her analysis of three newspapers in Kenya from 2002 to 2003, Omari found that 94% of all articles on women in the three dailies featured urban women as opposed to rural women (Omari, 2008). Further, in rural Kenya, limited access to computers and smartphones and unreliable electricity hinders online participation, especially for women (Wyche et al., 2013; Gustafsson, 2018). It is also possible that people outside the capital, where the parliamentarians reside, are relatively sheltered from information about the leaders who reside in the capital; thus, the national survey data might differ from the views of people exposed to information on the parliamentarians in the capital. We caveat this with our finding that the percentage of urban and rural men who agree men make better leaders is similar, but a higher percentage (∼2-3%) of rural women than urban women agree that men make better leaders (Afrobarometer, 2020).

Unlike most research on NLP and gender bias, we synthesized findings from an individual word embedding analysis and sentiment analysis for understanding the text. This approach could lead to a richer understanding of the text than possible through use of a single method, or using word embeddings as simply an input for a sentiment analysis. Sentiment analysis classifies the polarity and emotions of the language of a document. Most sentiment analysis studies of gender bias were previously limited to analyzing the sentiment of text from disparate female and male social media profiles or accounts. While there have been a few studies using sentiment analysis in Kenya, these have primarily been on social media with applications such as reactions to products/services or detecting terrorism. Analysis of gender bias and studies of news media are extremely limited and offer a different perspective than those based on social media (Gitau, 2011; Ngero, 2012, Ngoge, 2021). In this work, we used sentiment analysis to study the portrayal of female vs. male leaders within one media source, the *Daily Nation*, by separating and comparing sentences that include male vs. female leader names. Especially as female representation in leadership has increased from 2010 with the new Kenyan constitution, an understanding of the distribution of sentiments in the contextual text surrounding female and male leaders’ names provides unique insight into the media’s reactions and the connotations in which they may portray female leaders.

There are shortcomings of our analysis. Our results will to some extent depend on the wordlists chosen, including for the names and gender terms lists. Some leader names include common names that may not necessarily be connected to the political figure intended. However, we expect that these names are most likely to come up in the context of famous individuals mentioned in the news. Further, the names list will still provide information on the difference between men and women, even if some of the names are not referring to specific politicians. We tried to create sufficiently large wordlists (at least 30 words) for concepts in order to protect against an undue influence of word choice, but we may not have fully succeeded. We used linear trends in our analysis but the actual relationships we measured may not be linear. The sentiment predictor model was trained on the Stanford Sentiment Treebank, which is English text, so it may not fully account for dialect and language differences in our samples of Kenyan English media. Further, our method of separating the text into female and male inputs may have left out relevant sentences that were more than one sentence away from the leader name. Conversely, it may have led to the overlap of sentences that included both male and female leader names.

Off-the-shelf tools, such as AllenNLP, may not have been designed and/or trained optimally for all applications, such as investigating gender bias of Kenyan English. Additionally, word embeddings can be unstable, especially when trained on smaller datasets (see Supplementary Appendix 6 for our data sizes). Changes in parameter choice or data pre-processing can lead to potentially significant changes in embeddings, resulting in different conclusions with the same data. We completed “sensitivity” analyses on our results by testing both 1- and 3-year embeddings to evaluate consistency by data size. For pre-processing we followed conventions for GloVe and word2vec embeddings, such as moving to lowercase and removing punctuation.

For AllenNLP, due to the lack of availability of labeled sentiment data in Kenyan text, our model was trained on the English-based Stanford Sentiment Treebank (SST). The model achieved an accuracy rate of 0.80 on the SST training dataset and 0.41 on the validation set, which is comparable to the expected metrics for the realworldnlp LSTM model (Hagiwara, 2021). Our evaluation of the accuracy of the model more specifically with regard to Kenyan news media revealed an accuracy of 0.59. Finally, we conducted an additional analyses using AllenNLP’s more recently developed RoBERTa (Robustly optimized BERT pretraining approach) classification model. RoBERTa is pretrained on a very large training corpora, including over 63 million English news articles of various local dialects, and achieved state-of-the-art results so we expect RoBERTa to be robust in analyzing Kenyan English (Liu et al., 2019). The RoBERTa model when further trained on the binary classification setting of the SST achieved 0.95 accuracy on the validation set. We used this predictor to classify each of the scraped sentences per gender and year grouping as either having a negative sentiment (score of 0) or positive sentiment (score of 1). These further results indicated that text surrounding both female and male leaders had similar ranges of percent negative sentiment as our original AllenNLP model. The trends indicated slightly decreasing rates of negative sentiment and increasing rates of positive sentiment from 1998 to 2016 for both genders. The difference trend between female and male sentiments was not significant. The variation in sentiment trends and lack of a significant difference in the trends for male and female sentiments may result from RoBERTa’s binary classification setting, which loses the precision that our original AllenNLP LSTM model achieved with scores ranging from 0 to 4.

We did not replicate this analysis for another news media source in Kenya or elsewhere in East Africa, which is a logical next step. Use of newer embedding algorithms could be considered, including contextual language models, such as BERT, which have been created to take into account the context of words (Bhardwaj et al., 2020). These algorithms are popular for downstream tasks such as text classification and are also prone to learn intrinsic gender-bias. Looking into social media text (in English and Swahili) from blogs or social media sites such as Twitter could also be a next step. It is possible that our gender wordlists offer a skewed perspective for word counts and that women could be mentioned more by name. However, we found a large, 100 k differences in mentions of male and female words per year. This complements Nduva’s finding that prominent women leaders are not adequately covered by the media in Kenya (Nduva, 2016). In further support of this point, the Global Media Monitoring Project (GMMP), the largest and longest gender study on global media, has found that women are significantly underrepresented as presenters, reporters, and as news subjects in Kenyan news (Global Media Monitoring Project, 2015). However, the percentage of female reporters increased to 29 in 2015 from 13% in 2005 and mention of female news subjects increased to 22 from 9% of total news subjects (Global Media Monitoring Project, 2015).

### The Importance of the Media in Politics and Gender Equality

Our findings using NLP methods corroborate concerns expressed by women candidates in Kenya that they receive less media coverage than male candidates and when they are represented in the media they are put in a more negative light than men (National Democratic Institute and Federation of Women Lawyers [FIDA] Kenya, 2018). This corresponds to Omari’s finding in her study of East African newspapers from June 2002-2003 that in the *Daily Nation*, with a daily circulation at that time of about 300,000 copies [the highest circulation of the three dailies studied), articles on women constituted only 5% of the 3,101 articles analyzed (Omari, 2008). Other researchers have noted that media coverage of women leaders tends to highlight their appearance and personality traits while coverage of male leaders more often highlights their stances on issues or leadership ability (Kahn, 1994; Devitt, 2002; Bystrom, 2006; McIntosh, 2013; Nduva, 2016)]. This focus on women’s personality could lead to an evaluation of them based on likeability, with women being seen as “cold” or unlikeable if the article is overall negative (Bligh et al., 2012). On the other hand, focusing on a female leader’s ability could also result in dislike as her authority position challenges traditional feminine gender norms (Fiske et al., 2002; Casad, 2007; Bligh et al., 2012).

In the 2017 election, in response to negative coverage, women tended to avoid media-based public discourse and instead turned to social media to reach voters (National Democratic Institute and Federation of Women Lawyers [FIDA] Kenya, 2018). Thus, future applications of NLP could be extended to analysis of social media. Knowing the tendency of news media to portray women leaders negatively, some have argued that women politicians need to be more active in working with the media to try and stop negative stereotypes or negative coverage from proliferating (Tripp et al., 2014; Nduva, 2016; National Democratic Institute and Federation of Women Lawyers [FIDA] Kenya, 2018). More positively, women candidates overall thought that the quality of coverage of female politicians and candidates had improved significantly since 2013 (National Democratic Institute and Federation of Women Lawyers [FIDA] Kenya, 2018), which our research also corroborates.

Empirical research into whether female stereotypes harm female candidates offers conflicting conclusions. This may be because stereotype activation may be required before stereotypes influence perception of female politicians; therefore, when voters have minimal information, such as only the candidate’s political party, stereotypes will not be activated (Bauer, 2015). Stereotype activation could occur if women are mentioned in communal roles or framed in stereotypical ways, such as being depicted as nurturing (Caliskan et al., 2017) or associated with domestic activities as seen in our analysis. Framing is based on the idea that how a topic is presented in the news can influence how it is understood by audiences (Scheufele and Tewksbury, 2007). Frames of professional women in the media variably include the portrayal of women as a seductress or sex object, mothers, pets, or iron maidens, all of which undermine women (Carlin, 2009). This activation will magnify the incongruencies between the concepts of community (more stereotypically associated with women) and agency (more stereotypically associated with men), leading individuals to perceive women as poor leaders. Support of the women candidates will be diminished, especially at higher offices, where the most agentic characteristics are expected (Bauer, 2015).

## Conclusion

Word embeddings are a quantitative tool for measuring gender bias – one that could be used to compare the bias in multiple news sources or to track gender norms. Using a single newspaper, we found that at the level of Parliamentary leadership, women are portrayed more negatively than men. However, over time, this broad trend appears to be easing toward greater equality. Concerted actions may be able to further shape and accelerate this trend.

Word embeddings could be used as a monitoring and/or evaluation tool by NGOs and similar actors who are attempting to change viewpoints through such interventions. There are numerous organizations that have called for more inclusion of women in journalism and news media. NLP methods offer a way to help quantify progress or a lack thereof.

## Data Availability Statement

The datasets presented in this study can be found in online repositories: https://dataverse.harvard.edu/dataverse/kenyan-embeddings-and-sentiment and https://github.com/emmapair/Kenyan-Embeddings-and-Sentiment.

## Author Contributions

EP and NV: conceptualization, data curation, formal analysis, investigation, methodology, software, validation, visualization, and writing – original draft preparation. AW: conceptualization, funding acquisition, supervision, and writing – review and editing. VM: conceptualization, funding acquisition, methodology, software, supervision, and writing – review and editing. JZ: conceptualization, funding acquisition, methodology, and writing – review and editing. AN: conceptualization, resources, and writing – review and editing. GD: conceptualization, funding acquisition, project administration, resources, supervision, and writing – original draft preparation. All authors contributed to the article and approved the submitted version.

## Conflict of Interest

The authors declare that the research was conducted in the absence of any commercial or financial relationships that could be construed as a potential conflict of interest.

## Publisher’s Note

All claims expressed in this article are solely those of the authors and do not necessarily represent those of their affiliated organizations, or those of the publisher, the editors and the reviewers. Any product that may be evaluated in this article, or claim that may be made by its manufacturer, is not guaranteed or endorsed by the publisher.

## Supplementary Material

Supplementary Appendix 1 One-year bias for leader words related to gender words. A negative bias means the leader word list is more associated with men.

Supplementary Appendix 2 Afrobarometer survey results on gender and leadership. Separated by **(A)** Male education levels **(B)** Female education levels and **(C)** whether respondents were urban or rural.

Supplementary Appendix 3 One-year bias for gender words and leader names and word lists for adjectives related to leadership. For **(A)** Corruption; **(B)** Defamation; **(C)** Determination; **(D)** Intelligence; **(E)** Competence; **(F)** Good; **(G)** Rationality; **(H)** Domestic; **(I)** Immorality; **(J)** Influence; and **(K)** Bad Leader.

Supplementary Appendix 4 Regression results.

Supplementary Appendix 5 Gender noun counts over time. The count of male and female gender words in *Daily Nation* article text from 1998 to 2019.

Supplementary Appendix 6 Kenya’s *Daily Nation* article counts by year used in natural language processing analyses.
