# Supplementary figures and images for: Quantification of Gender Bias and Sentiment Toward Political Leaders Over 20 Years of Kenyan News Using Natural Language Processing
Source: Front Psychol. 2021 Dec 10;12:712646. doi: 10.3389/fpsyg.2021.712646 (PMC8703202; doi:10.3389/fpsyg.2021.712646)

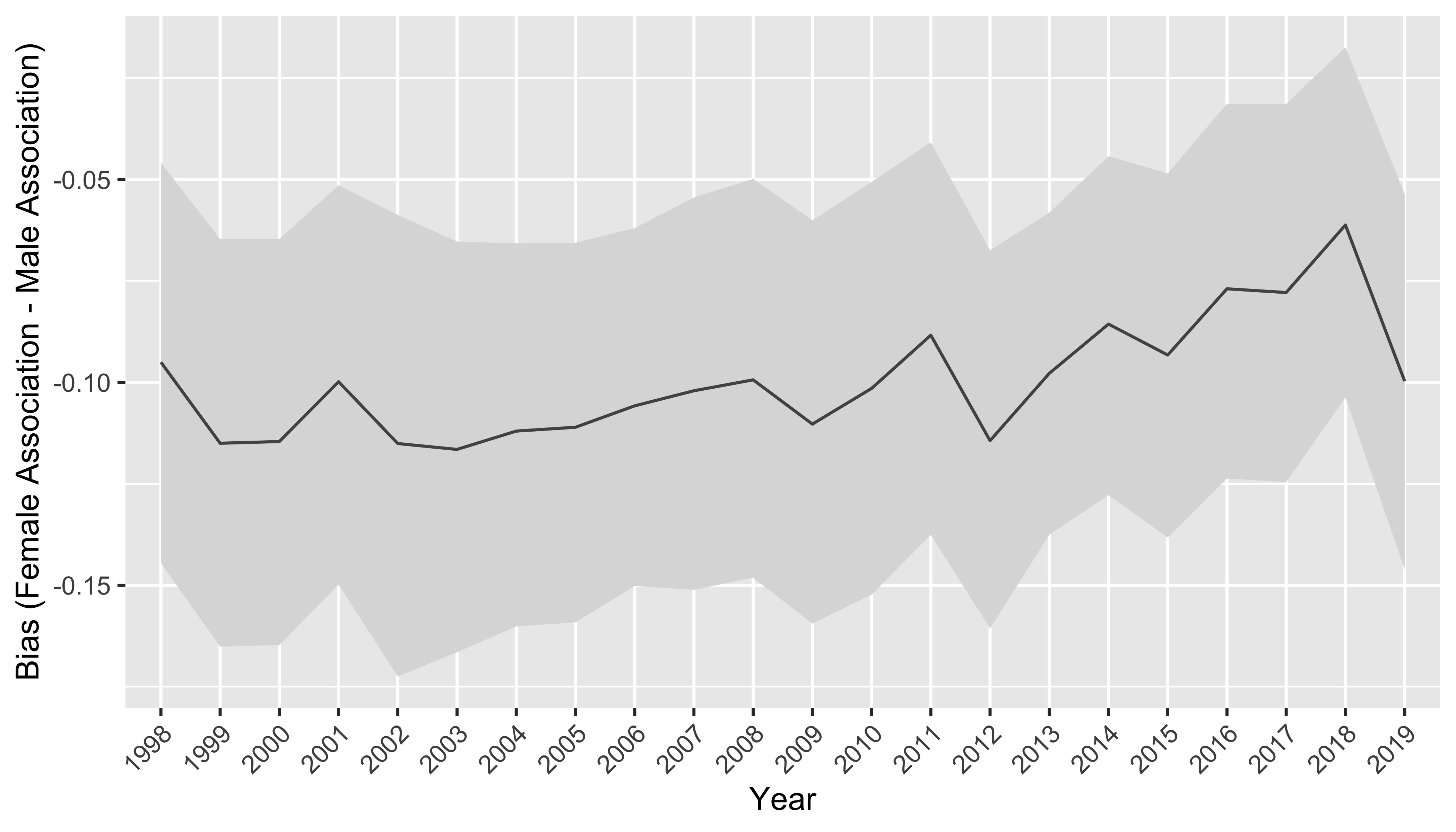

Supplement: Supplementary Appendix 1 — One-year bias for leader words related to gender words. A negative bias means the leader word list is more associated with men. [file Image_1.JPEG]

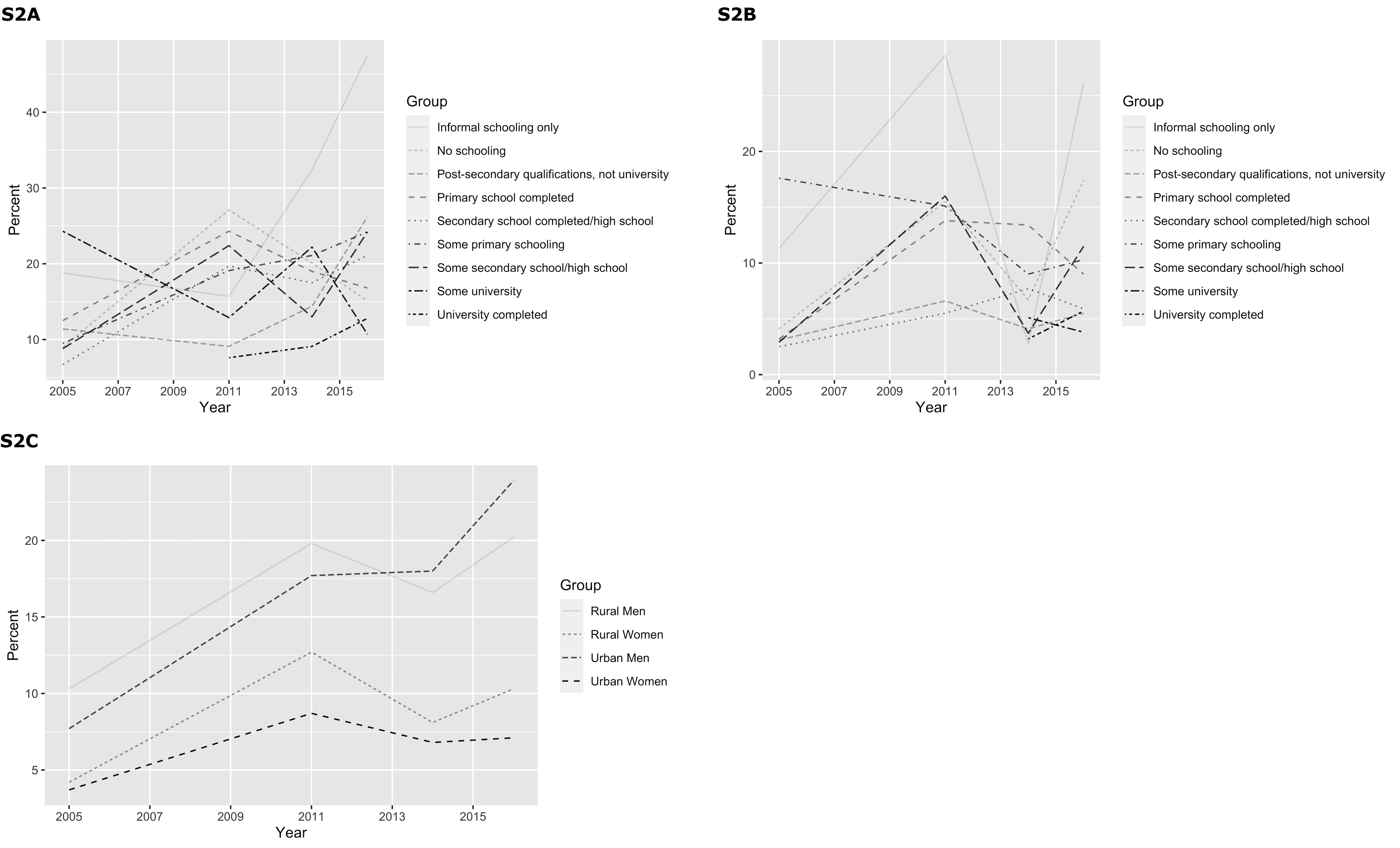

Supplement: Supplementary Appendix 2 — Afrobarometer survey results on gender and leadership. Separated by (A) Male education levels (B) Female education levels and (C) whether respondents were urban or rural. [file Image_2.JPEG]

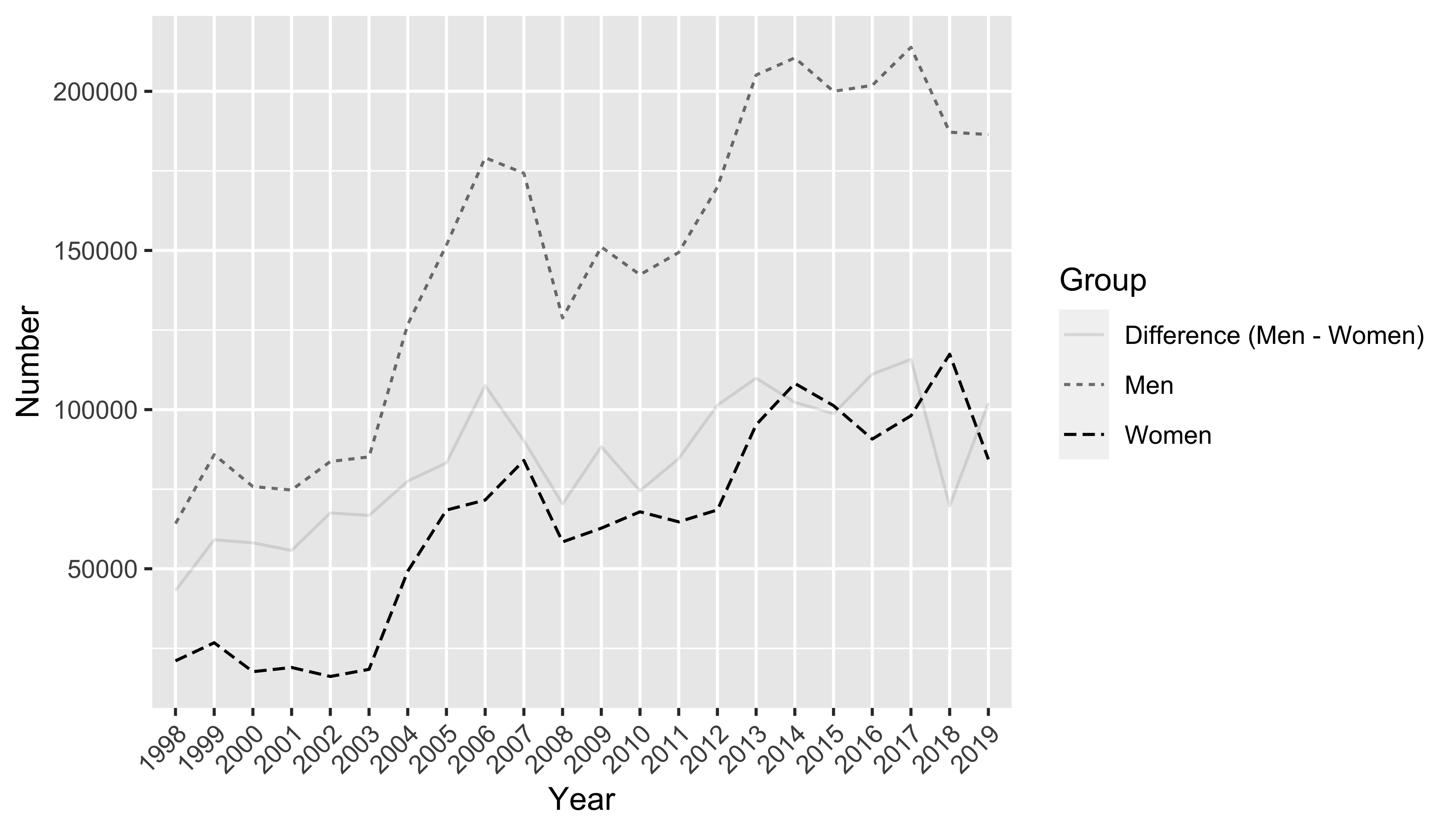

Supplement: Supplementary Appendix 3 — One-year bias for gender words and leader names and word lists for adjectives related to leadership. For (A) Corruption; (B) Defamation; (C) Determination; (D) Intelligence; (E) Competence; (F) Good; (G) Rationality; (H) Domestic; (I) Immorality; (J) Influence; and (K) Bad Leader. [file Image_3.JPEG]

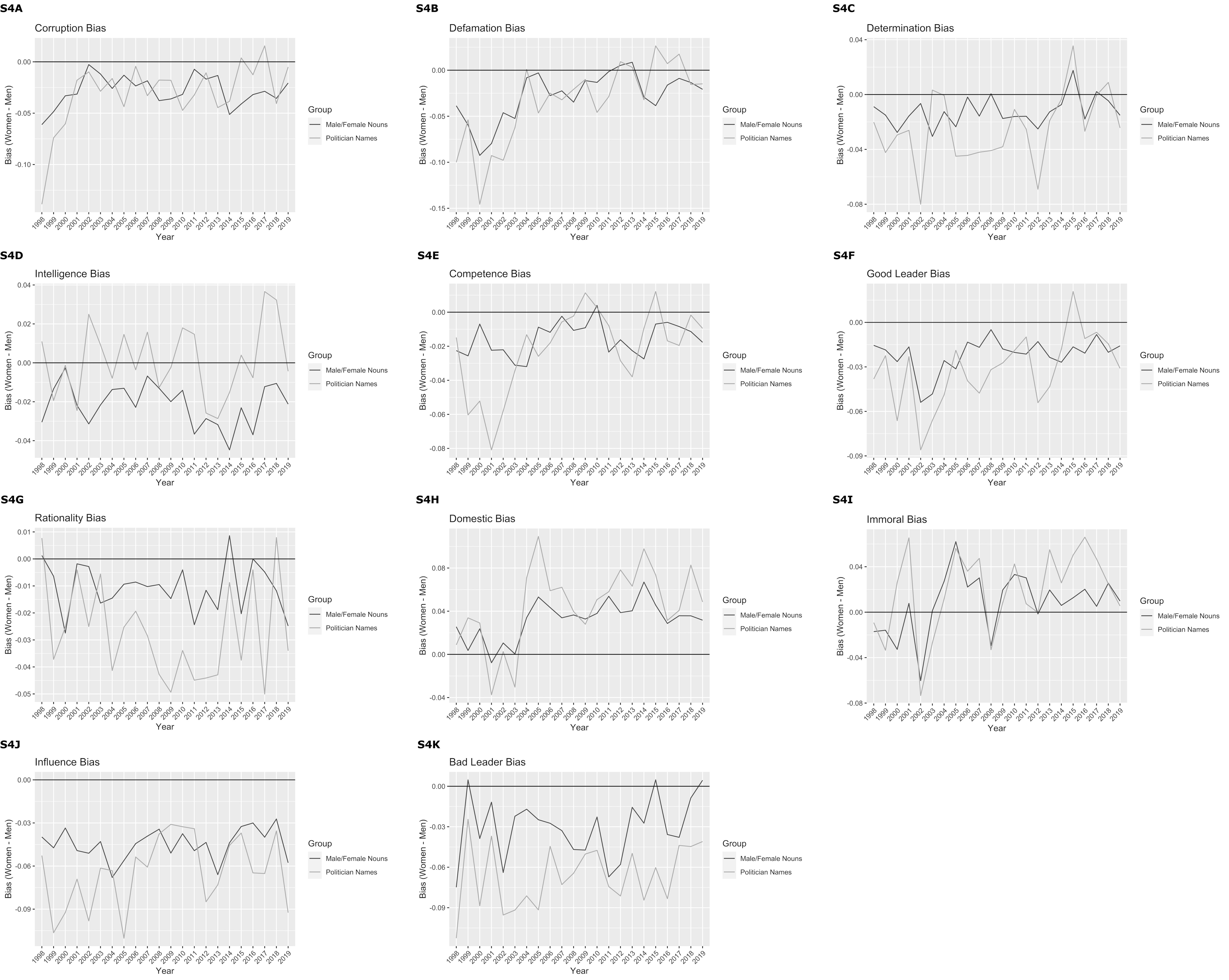

Supplement: Supplementary Appendix 4 — Regression results. [file Image_4.JPEG]

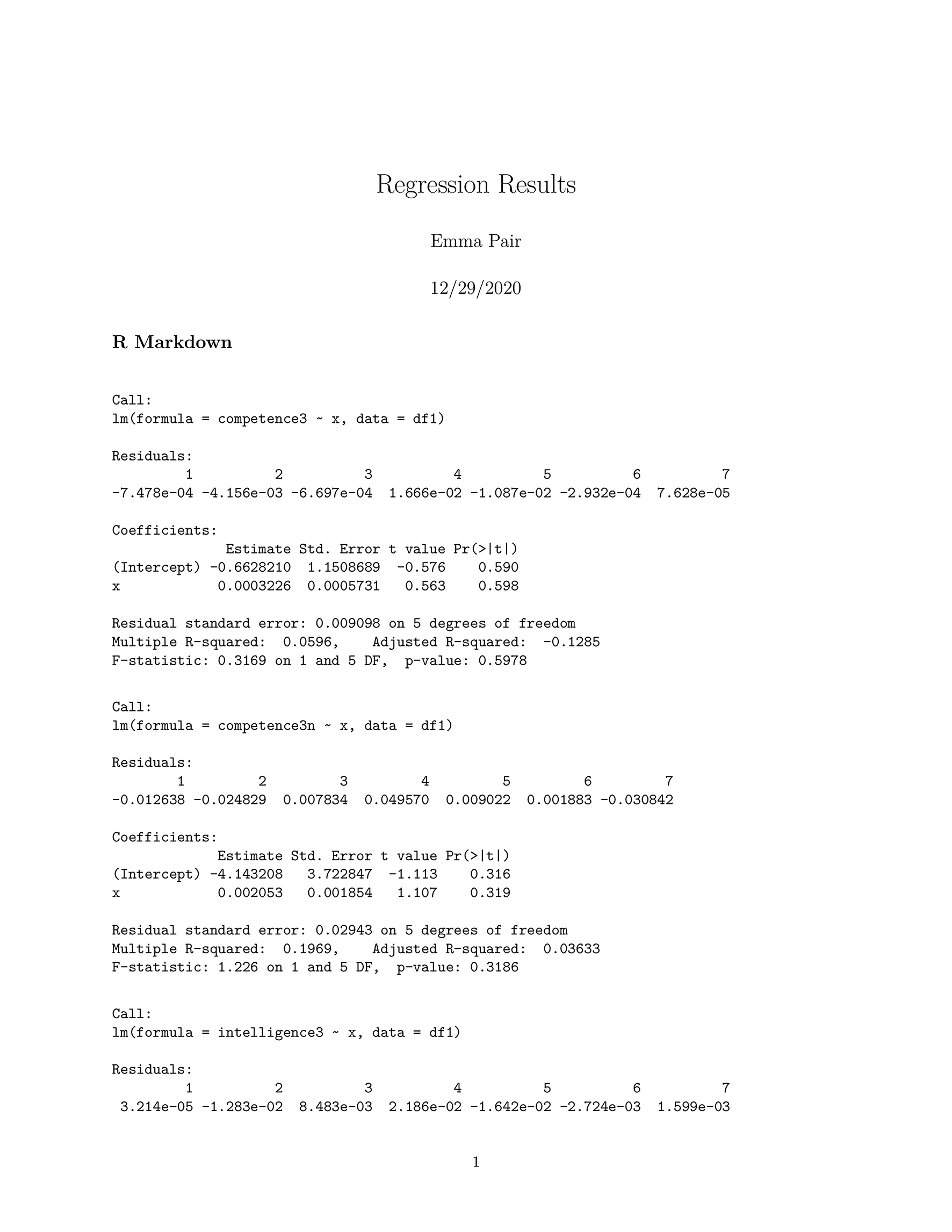

Supplement: Supplementary Appendix 5 — Gender noun counts over time. The count of male and female gender words in Daily Nation article text from 1998 to 2019. [file Image_5.TIF]

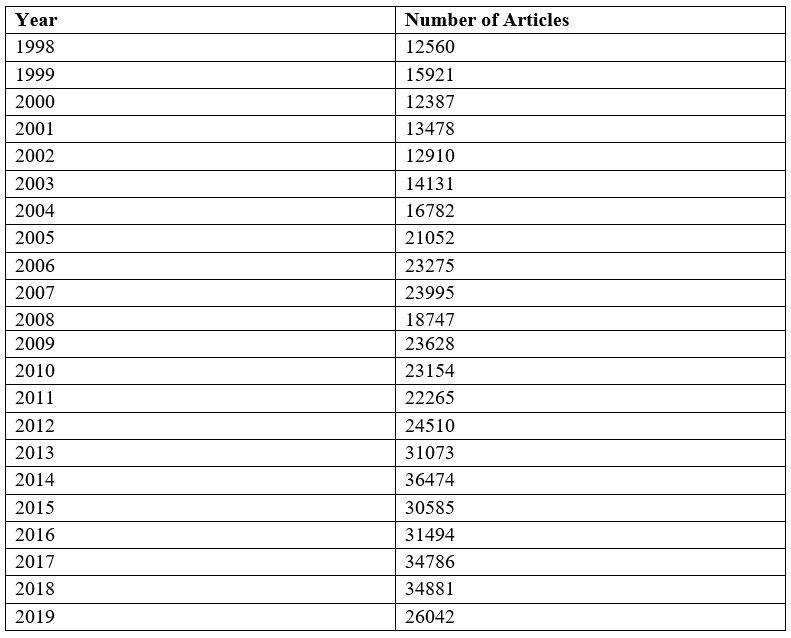

Supplement: Supplementary Appendix 6 — Kenya’s Daily Nation article counts by year used in natural language processing analyses. [file Image_6.JPEG]
